# Challenging microalgal vitamins for human health

**DOI:** 10.1186/s12934-020-01459-1

**Published:** 2020-11-02

**Authors:** Angelo Del Mondo, Arianna Smerilli, Elisabet Sané, Clementina Sansone, Christophe Brunet

**Affiliations:** Stazione Zoologica Anton Dohrn, Istituto Nazionale Di Biologia, Ecologia e Biotecnologie marine, Villa Comunale, 80121 Napoli, Italy

**Keywords:** Vitamin D, Vitamin K, Microalgae, Biotechnology, Antioxidants, Nutraceuticals

## Abstract

**Background:**

Vitamins’ deficiency in humans is an important threat worldwide and requires solutions. In the concept of natural biofactory for bioactive compounds production, microalgae represent one of the most promising targets filling many biotechnological applications, and allowing the development of an eco-sustainable production of natural bioactive metabolites. Vitamins are probably one of the cutting edges of microalgal diversity compounds.

**Main text:**

Microalgae can usefully provide many of the required vitamins in humans, more than terrestrial plants, for instance. Indeed, vitamins D and K, little present in many plants or fruits, are instead available from microalgae. The same occurs for some vitamins B (B_12_, B_9_, B_6_), while the other vitamins (A, C, D, E) are also provided by microalgae. This large panel of vitamins diversity in microalgal cells represents an exploitable platform in order to use them as natural vitamins’ producers for human consumption. This study aims to provide an integrative overview on vitamins content in the microalgal realm, and discuss on the great potential of microalgae as sources of different forms of vitamins to be included as functional ingredients in food or nutraceuticals for the human health. We report on the biological roles of vitamins in microalgae, the current knowledge on their modulation by environmental or biological forcing and on the biological activity of the different vitamins in human metabolism and health protection.

**Conclusion:**

Finally, we critically discuss the challenges for promoting microalgae as a relevant source of vitamins, further enhancing the interests of microalgal “biofactory” for biotechnological applications, such as in nutraceuticals or cosmeceuticals.

## Background

The class of vitamins includes a diversity of organic compounds that represent essential micro-nutrients for life. These molecules cover a plethora of biological functions, such as coenzymes, hormones, antioxidants, mediators of cell signalling and regulators of cell and tissue growth or differentiation. Vitamins can be divided in two large groups, the water-soluble and fat-soluble compounds. Vitamins A, D, E and K are the four fat-soluble molecules, while the vitamin C and the vitamins B [B_1_ (thiamin), B_2_ (riboflavin), B_3_ (niacin = nicotinic acid), B_5_ (pantothenic acid), B_6_ (pyridoxine), B_7_ (biotin), B_9_ (folic acid) and B_12_ (cobalamin)] are water-soluble. Most of the vitamins are synthetized by photosynthetic organisms, while others (some vitamins B and vitamin K) are bioaccumulated through diet and mainly produced by bacteria [1]. Accumulation and/or synthesis of vitamins in photosynthetic organisms is highly variable [2], and strongly related to physiological responses to environmental cues [3]; the magnitude of these responses being dependent on the fitness between organism and the environment [4].

Although crucial for life, vitamins are either not or little synthetized in animals and humans, thus requiring their continuous assimilation through diet, e.g. from plants, fruits or seeds. In order to avoid vitamins deficit in humans, it is strongly recommended to follow diets with high content in the different vitamins. However, not all plants contain all vitamins, and some of them (vitamins D, K or some B) are scarcely present.

**Fig. 1:**
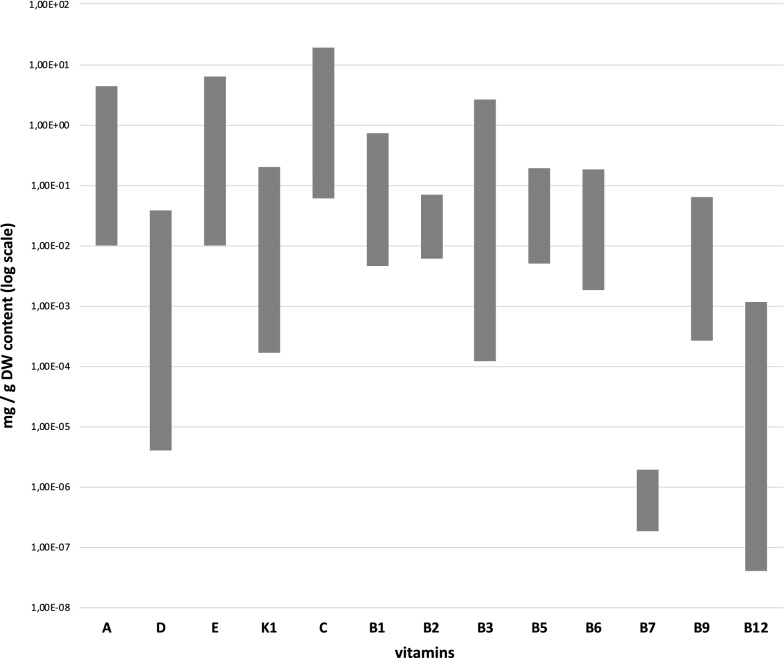
Vitamin content (mg g^−1^ of dry weight biomass) variability in microalgae. Axis Y in logarithmic scale

Among plant kingdom, marine algae produce or/and accumulate a large diversity of vitamins (Fig. 1), and microalgae—photosynthetic unicellular and fast dividing rate organisms—potentially could be extremely helpful as vitamins’ producers, as the already known “super food” vitamins-rich *Spirulina platensis* [5]. Microalgae can contain vitamins such as vitamins B_12_ [3, 6], vitamin K [7] or D [8] that are not present in higher plants. Vitamin D is known to be highly concentrated in sea edible organisms (e.g., fishes, [9]) which accumulate it through algal based diets, being not able to synthetize it [10]. The content in the other vitamins, generally provided by higher plants, can be also significant if we consider that are unicellular organisms. For instance, the green microalga *Dunaliella* is known to highly accumulate the vitamins B_2_, B_12_, B_9_, B_3_ as well as the vitamins C and E [11] while high content of vitamin C has been reported in the diatom *Skeletonema marinoi* [12, 13].

The general aim of this study is to critically discuss if and why microalgae can become a functional source of vitamins to fill human requirements through food complements or nutraceuticals. In biotechnology, microalgae can be defined a real and natural biofactory of bioactive compounds as well as vitamins for dietary intake [14–16]. It is noteworthy that some vitamins (e.g., vitamins E, A or C) have been the object of numerous studies, although other vitamins were poorly investigated in microalgae. Here, we update an integrated state-of-art on the vitamins A, D, E, K, C, B_1_, B_2_, B_3_, B_5_, B_6_, B_7_, B_9_ and B_12_ contents in microalgae, as well as on their biological roles and modulation by environmental or biological forcing in microalgae. We also report on the biological activity of the different vitamins in human metabolism and health protection.

## Functions and roles of vitamins in microalgae

In living organisms, vitamins are involved in numerous processes and functions, being required for example as precursors or coenzymes in key metabolic pathways, controlling and regulating tissue growth and cell functioning, or acting as antioxidants. Some vitamins have few specific biological functions (e.g., vitamin B_7_), while others display multiple roles (e.g., vitamin C).

In microalgae, **vitamin A** is mainly synthetized from the provitamin A carotenoids (e.g., β-carotene, β-cryptoxanthin, and α-carotene) [17]. The well-known β–carotene is the precursor of many carotenoids, such as those belonging to the photoprotective xanthophyll cycle (Arianna [13] and acts as antioxidant in photosynthetic organisms [17]. Indeed, the efficacy of β-carotene against the reactive oxygen species singlet oxygen is greater than vitamin E and vitamin C [18].

The involvement of the **vitamins D**_**2**_** and D**_**3**_ in cellular functions in microalgae remains unclear. In higher plants that are able to synthesize vitamin D_2_, the latter exerts a role as a growth factor [19]. Provitamin D has been hypothesized to act as a UV-B receptor in plants [19]. It has been speculated that the vitamin D: provitamin D ratio might be a proxy of UV-B assimilation in plants [20]. In microalgae, vitamin D production might be the result of damage or degradation of biological membranes under the action of UV radiation [21, 22].

**Vitamin E** presents roles as antioxidant or also against photooxidative stress [23]. When tocopherol synthesis is chemically blocked in algae exposed to high light, in association with PS II inactivation, the tocopherol pool undergoes to rapid depletion due to its action against photooxidative stress [24]. It has been hypothesized a complementary role of tocopherol with the photoprotective xanthophyll cycling-pigments [25]. Other studies showed that in microalgae tocopherol production is often associated with polyphenols production in response to abiotic stress, such as light, nutrient or metals [26–28].

**Vitamin K**_**1**_ has a function of redox cofactor in plants, green algae and some cyanobacteria [29–31]. Specifically, vitamin K_1_ is the secondary electron acceptor of photosystem (PS I), also known as A1 [32]. However, it is now accepted that at least half of vitamin K_1_ is not bound to PS I [33, 34], suggesting a role of phylloquinone in redox reactions distinct from that of the one-electron transfer in PS I. Likewise, menaquinone (**vitamin K**_**2**_) is a secondary electron acceptor of PS I in red algae, diatoms, cyanobacteria and archaeal species [35–38].

**Vitamin C** acts as a cofactor of many enzymes, and is involved in photosynthesis, hormone biosynthesis and regeneration of antioxidants [39, 40]. Ascorbic acid plays a relevant role in algal photoprotection, being used as a co-factor for the de-epoxidase enzyme operating in the photoprotective xanthophyll cycle (violaxanthin-antheraxanthin-zeaxanthin or diadinoxanthin-diatoxanthin) for the synthesis of the photoprotective xanthophyll [13, 41]. Indeed, in the diatom *Skeletonema marinoi*, high intensity blue light induced a parallel increase of ascorbic acid and xanthophyll cycle activity [13]. Inside the chloroplast, ascorbic acid plays a key role in photosynthesis by removing hydrogen peroxide formed by oxygen photoreduction in PSI (Mehler reaction) via ascorbate peroxidase [42]. In coordination with glutathione and enzymatic antioxidants in chloroplasts, mitochondria, peroxisomes and cytosol, ascorbic acid controls the amount of hydrogen peroxide formed within the cell [43]. The expression of the VTC2 gene (GDP-L-galactose phosphorylases catalyzing the first step in the L-ascorbate biosynthesis) in *Chlamydomonas reinhardtii* is rapidly induced by hydrogen peroxide and singlet oxygen: the subsequent response, resulting in a manifold increase in ascorbate content, conversely to plants does not require circadian regulation nor photosynthesis [44].

Although in plants ascorbic acid controls a number of processes including cell division and cell expansion [17, 18], this role is not confirmed in microalgae, at the exception of a study in red algae [45].

**Vitamin B**_**1**_ is ubiquitously involved in the acetyl-CoA synthesis, tricarboxylic acid cycle, pentose phosphate pathway, Calvin–Benson cycle and isoprenoid biosynthesis pathway [46]. This vitamin is known to exert a defense function against abiotic and biotic stressors in plants [46] and in several microalgae [47]. A putative role of vitamin B_1_ as antioxidant has been hypothesized [48], even though the mechanism of this potential function remains unknown.

**Vitamin B**_**2**_ is an essential precursor for flavocoenzymes, involved in numerous physiological processes such as the circadian clock [25, 49–51], or acting as chromophores in blue light photoreceptors of plants and fungi [52–54]. Flavocoenzymes can catalyze redox processes involving one- and two-electron transitions as well as a variety of reactions such as photorepair of thymidine dimers in photodamaged DNA [55, 56].

**Vitamin B**_**3**_ is required for assimilatory nitrate reductase (NR) activity in photoautrotrophs [57, 58], beside its physiological role under the form of NADH [58]. Niacin vitamers—nicotinic acid, nicotinamide, NAD, and NADP—contribute to the antioxidant cell machinery. The antioxidant enzyme monodehydroascorbate reductase can use either NADH or NADPH [59], reduced by the plant mitochondrial electron transport chain [60].

**Vitamin B**_**5**_ is synthesized de novo by plants and micro-organisms. It is involved in many secondary metabolite biosynthetic pathways [61] and acts as precursor of the 40-phosphopantetheine moiety of coenzyme A (CoA) and acyl carrier protein. Due to the crucial role of the CoA in central carbohydrate and lipid metabolism, vitamin B_5_, synthesized in the cytosol, is subsequently transported into mitochondria and plastids [61].

**Vitamin B**_**6**_ is a cofactor for numerous metabolic enzymes [62] and acts as a potent antioxidant [4, 62] quenching efficiently reactive oxygen species [36, 63] with an efficacy comparable to ascorbic acid and α-tocopherol [64, 65]. Recently, a role of vitamin B_6_ in UV-B leaf acclimation has been demonstrated in plants, showing that vitamin B_6_ deficient *Arabidopsis thaliana rsr4-1* mutant cannot cope with supplementary UV-B radiation [66].

**Vitamin B**_**7**_ is a cofactor for some carboxylases, decarboxylases and transcarboxylases involved in metabolic processes such as fatty acid and carbohydrate metabolism [67].

**Vitamin B**_**9**_ is an essential cofactor for one-carbon metabolism and primarily for the synthesis of the purine ring [68]. In algae, that are able to accumulate high concentrations of folates [69], folate biosynthetic route has recently been elucidated, showing that algae possess single isoforms of the genes in the pathway, while plant species tend to have multiple isoforms regulating the same steps in folate metabolism [70].

**Vitamin B**_**12**_ is involved in two core enzymatic reactions in algae, the DNA synthesis—being the cofactor of a form of the enzyme methionine synthase, and the inorganic carbon assimilation being required as a cofactor by the enzyme methylmalonyl CoA mutase [71].

## Content and modulation of vitamins in microalgae

### Vitamin A content

Vitamin A in microalgae varied from 0.01 mg per gram of dry weight (mg/g DW) as reported in the genera *Chlorella* and *Isochrysis* to 4.28 mg/g DW reported in the genus *Tetraselmis* (Table 1).

**Table 1:** Vitamin A content in microalgae

Phylum/class	Genus	Vitamin A	Refs
Cyanobacteria	*Anabaena*	0.28	[72]
	*Arthrospira*	0.65	[73]
	*Synechococcus*	0.18	[73]
Chlorophyta	*Chlamydomonas*	0.11–0.13	[72]
	*Chlorella*	0.01–0.65	[72, 74, 75]
	*Dunaliella*	0.01–0.63	[74, 76]
	*Stichococcus*	0.06	[77]
	*Tetraselmis*	0.05–4.28	[73, 74, 77, 78]
Rhodophyta	*Porphyridium*	0.75	[73]
Bacillariophyceae	*Chaetoceros*	0.52–0.97	[73, 78]
	*Skeletonema*	0.14	[78]
Haptophyta	*Isochrysis*	0.01–0.27	[74, 78]
	*Pavlova*	0.10 -0.26	[77, 78]
Eustigmatophyceae	*Nannochloropsis*	0.05–0.08	[73, 77]
Euglenozoa	*Euglena*	0.30	[79]

Values are expressed as mg/g DW of retinol equivalents

Low vitamin A content was recorded in *Nannochloropsis* (ranging between 0.05 and 0.08 mg/g DW), while the latter displays high content of vitamins C and E, and in the euglenoid *Euglena* (0.30 mg/g DW) and in *Pavlova* (0.27 mg/g DW). Vitamin A content generally ranged between 0.50–0.80 mg/g DW (Table 1), such as in the cyanophyte *Arthrospira*, or in the green microalgae *Chlorella* and *Dunaliella* (Table 1). High vitamin A concentration in diatoms were reported in the genus *Chaetoceros* (from 0.52 to 0.97 mg/g DW), as well as in the red microalga *Porphyridium* (0.75 mg/g DW, Table 1). Vitamin A content strongly varied among and inside algal classes hypothesizing that no link between vitamin A concentration and microalgal divisions do exist (Table 1). Using a conversion factor between dry and fresh weight for microalgae of around 10% [80], it can be estimated a content of 0.42 and 0.1 mg of retinol equivalents per g of fresh weight (mg RE/g FW) in *Tetraselmis* and *Chaetoceros*. These values are much higher than those reported in edible carrot (*circa* 0.011 mg RE/ g FW) or in orange (0.0003 mg RE/g FW) [81].

### Vitamin C content

Vitamin C content in microalgae varied between 0.06 and 18.79 mg/g DW (Table 2), displaying a great inter and intra specific variability. Indeed, in green microalga *Chlorella*, ascorbic acid content ranged from 0.10 to 15 mg/g DW (Table 2). Yet, a variation from 0.16 to 2.20 mg/g DW ascorbic acid was reported in the genus *Dunaliella* (Table 2). Diatoms displayed a great variability in vitamin C content, ranging from 0.06 to 6.7 mg/g DW in *Skeletonema*, or from 0.12 to 18.79 mg/g DW in *Chaetoceros* (Table 2). Large variability was also reported in haptophytes (Table 2), while ascorbic acid content was high in *Nannochloropsis*, with values ranging from 2.50 to 6.04 mg/g DW (Table 2). Values around 2 mg/g DW of ascorbic acid were recorded in the green microalga *Scenedesmus*, the cyanophyte *Anabaena,* the cryptophyte *Chroomonas* and the euglenoid *Euglena* (Table 2). Using the 10% conversion factor between DW and FW [80], microalgal vitamin C can reach concentrations of 1.88 mg/g FW (*Chaetoceros*), 1.5 mg/g FW (*Chlorella*) or 0.6 mg/g FW (*Nannochloropsis*) in the range or higher than some vitamin C-rich fruits, as strawberries, kiwis or lemons (0.54, 0.52 and 0.42 mg/g FW, respectively [82]).

**Table 2:** Vitamin C content in microalgae

Phylum/class	Genus	Vitamin C	Refs
Cyanobacteria	*Anabaena*	2.00	[72]
Chlorophyta	*Chlamydomonas*	2.00	[72]
	*Chlorella*	0.10–15	[72, 74, 75]
	*Dunaliella*	0.16–2.2	[74, 76, 83]
	*Nannochloris*	5.24	[83]
	*Scenedesmus*	2.00	[72]
	*Stichococcus*	2.50	[77]
	*Tetraselmis*	0.19–3	[74, 77, 78]
Bacillariophyceae	*Chaetoceros*	0.12–18.79	[78, 83]
	*Skeletonema*	0.06–6.7	[13, 78, 83]
	*Thalassiosira*	1.79	[83]
Haptophyta	*Isochrysis galbana*	0.12–4.45	[74, 78, 83]
	*Pavlova lutheri*	0.84–1.3	[77, 78, 83]
Eustigmatophyceae	*Nannochloropsis*	2.50–6.04	[77, 83]
Ochromonadaceae	*Chroomonas*	2.13	[83]
Euglenozoa	*Euglena gracilis*	1.82	[79]

Values are expressed as mg/g DW of ascorbic acid

### Vitamin E content

Vitamin E concentration in microalgae ranged between 0.01 and 6.32 mg/g DW (Table 3). High vitamin E content was found within the genera *Tetraselmis* (6.32 mg/g DW), *Chlamydomonas* (4 mg/g DW), *Chlorella* (2 mg/g DW) and *Dunaliella* (1.90 mg/g DW). Among cyanobacteria, high values were reported for the genera *Anabaena* (4 mg/g DW), and *Arthrospira* (2.50 mg/g DW) or *Synechococcus* (1.40 mg/g DW).

**Table 3:** Vitamin E content in microalgae

Phylum/class	Genus	Vitamin E	Refs
Cyanobacteria	*Anabaena*	4	[72]
	*Aphanizomenon*	0.10–0.14	[84]
	*Arthrospira*	0.11–2.50	[73, 84]
	*Oscillatoria*	0.09–0.10	[84]
	*Synechococcus*	1.40	[73]
	*Synechocystis*	0.17	[84]
Chlorophyta	*Asterochloris*	0.09	[84]
	*Botrydiopsis*	0.06–0.17	[84]
	*Botryococcus*	0.16–0.26	[84]
	*Bracteacoccus*	0.17	[84]
	*Chlamydomonas*	0.34–4	[72, 84]
	*Chlorella*	0.01–2	[72, 74, 84–86]
	*Chlorellidium*	0.69	[84]
	*Chloridella*	0.04	[84]
	*Chlorococcum*	0.79	[84]
	*Chloroidium*	0.32	[84]
	*Chloromonas*	0.41–0.7	[84]
	*Chorycystis*	0.26	[84]
	*Chromochloris*	0.18	[84]
	*Coccomyxa*	0.66	[84]
	*Coelastrella*	0.42–0.51	[84]
	*Coelastrum*	0.07	[84]
	*Coenochloris*	0.74	[84]
	*Coenocystis*	0.36	[84]
	*Coleochlamys*	0.37	[84]
	*Desmodesmus*	0.19–0.39	[84, 85]
	*Dunaliella*	0.12–1.9	[74, 76, 85, 87]
	*Edaphochlorella*	0.24	[84]
	*Enallax*	0.02	[84]
	*Fottea*	0.48	[84]
	*Geminella*	0.01–0.08	[84]
	*Haematococcus*	0.27–0.88	[84]
	*Heterochlorella*	0.01	[84]
	*Interfilum*	0.05	[84]
	*Klebsormidium*	0.06–0.09	[84]
	*Lobosphaeropsis*	0.10	[84]
	*Monodopsis*	0.46	[84]
	*Monodus*	0.32–0.5	[84]
	*Muriella*	0.62	[84]
	*Neocystis*	0.38	[84]
	*Neospongiococcum*	0.06	[84]
	*Pabia*	0.36	[84]
	*Pectinodesmus*	0.03	[84]
	*Pseudobumilleriopsis*	0.18	[84]
	*Pseudochlorella*	0.05–0.15	[84]
	*Pseudomuriella*	0.24	[84]
	*Scenedesmus*	0.08–1	[72, 84]
	*Scotiellopsis*	0.44	[84]
	*Stichococcus*	0.13–0.44	[84]
	*Tetradesmus*	0.05–0.13	[84]
	*Tetraedron*	0.12–0.22	[73, 74, 77, 84, 87, 88]
	*Tetraselmis*	0.04–6.32	[78]
	*Trebouxia*	0.07–0.14	[84]
	*Trentepohlia*	0.28	[84]
Rhodophyta	*Porphyridium*	0.02–1.30	[73, 84]
	*Rhodella*	0.03–0.07	[84]
Bacillariophyceae	*Chaetoceros*	0.89–1.63	[73, 78]
	*Phaeodactylum*	0.01	[85]
	*Skeletonema*	0.11	[78]
Haptophyta	*Diacronema*	0.40	[89]
	*Isochrysis*	0.06–0.12	[74, 78]
	*Pavlova*	0.14–0.35	[78]
Eustigmatophyceae	*Microchloropsis*	0.23–0.67	[84]
	*Nannochloropsis*	0.02–4.72	[73, 77, 84, 85, 88, 90]
Xanthophyceae	*Heterococcus*	0.09–0.22	[84]
	*Xanthonema*	0.16–0.39	[84]
	*Vischeria*	0.04–0.05	[84]
Euglenozoa	*Euglena*	0.28–1.2	[79, 91]

Values are expressed as mg/g DW of α-tocopherol

High α-tocopherol concentration variability was described in *Nannochloropsis* (0.02–4.72 mg/g DW) and *Porphyridium* (0.02–1.30 mg/g DW). Low values were reported in the xanthophyceans *Heterococcus*, *Xanthonema* and *Vischeria* (0.04–0.39 mg/g DW), and in the red microalga *Rhodella* (0.03–0.07 mg/g DW) as well as in the haptophytes *Diacronema, Isochrysis* and *Pavlova* or in diatoms, with the exception of *Chaetoceros* (1.63 mg/g DW). Using the 10% conversion factor between DW and FW [80], *Tetraselmis*, *Nannochloropsis* and *Anabaena* reached 0.63, 0.40 and 0.48 mg/g FW of α-tocopherol, respectively. These values are notably higher than the vitamin E content of common dietary food, e.g. 0.03 mg/g FW for green olives, 0.02 mg/g FW for raw spinaches and 0.01 mg/g FW for blackberries and cranberries [92].

### Vitamins B content

Among the microalgal vitamins B data, vitamins B_1_ and B_12_ were the most studied (Table 4).

**Table 4:** Vitamins B content in microalgae (μg/g DW except for vitamins B_7_ and B_12_ in ng/g DW)

Phylum/class	Genus	Vitamin B_1_	Vitamin B_2_	Vitamin B_3_	Vitamin B_5_	Vitamin B_6_	Vitamin B_7_	Vitamin B_9_	Vitamin B_12_	Refs
Cyanobacteria	*Anabaena*	5.8	55	78	88	7	0.18	15	1.5	[72, 94]
	*Aphanizomenon*	40	6	130	8	13		1	6	[95]
	*Arthrospira*	10–23.8	33–45	0.13–149	13	9.6		0.27–4.8	0.50–6.6	[6, 94, 95]
Chlorophyta	*Chlamydomonas*						0.26	9		[72]
	*Chlorella*	18–23	20–68	0.15–250	21.4–190	1.9–25	0.45–1.1	3.1–34	0.08–2.5	[6, 69, 72, 75, 95]
	*Dunaliella*	9–29	9–31.2	10	5–13.2	2.2–4	0.9	0.4–53.7	0.04–0.7	[69, 74, 95]
	*Haematococcus*	4.7	17	66	14	3.6		2.9	1.2	[95]
	*Picochlorum*							64.7		[69]
	*Stichococcus*	29	25			17	1.3	24	1.95	[77]
	*Tetradesmus*							25.9		[69]
	*Tetraselmis*	32.3–627	19.1–42	1410	37.7	2.8–155	0.8–1.3	3–20	1.95–9	[74, 77, 78]
	*Scenedesmus*		46					6		[72]
Rhodophyta	*Porphyridium*							5.39		[69]
Bacillariophyceae	*Chaetoceros*	655	12	25		4			8	[78]
	*Skeletonema*	710	37	511		134			117	[78]
Haptophyta	*Isochrysis*	14–462	14–30	2690	9.1	1.8–183	1	3	0.6–89	[74, 78]
	*Pavlova*	36–290	6–50	955		4–8.4	1.9	23	1.7–1162	[77, 78]
Eustigmatophyceae	*Microchloropsis*							43.6		[69]
	*Nannochloropsis*	70	22–25	0.12		3.6	1.1	17–22	0.3–1.7	[6, 77]
Ochromonadaceae	*Poteriochromonas*		27.57			4.86				[96]
Euglenozoa	*Euglena*		55.71			14.71	0.22			[96]

Diatoms and Haptophytes displayed higher average concentrations of vitamins B_1_ and B_12_ compared to Chlorophyta and Cyanobacteria (Table 4), with the highest B_12_ concentration (1.17 ng/g DW) reported in the haptophyte *Pavlova* (Table 4). High B_12_ contents were also revealed in the cyanobacteria *Aphanizomenon* and *Arthrospira* as well as in the green alga *Chlorella* (Table 4). Notably, microalgae can reach high concentration of vitamins B_2_, B_3_ and B_6_, as in the cyanobacterium *Aphanizomenon* or in the haptophyte *Pavlova* (Table 4). Vitamin B_6_ content was reported to be high in several macroalgae [93], as well as in microalgae (Table 4). A wide range of vitamin B_6_ concentrations has been reported for the green microalga *Tetraselmis* and for the haptophyte *Isochrysis*, with values from 2.8 to 155 μg/g DW and from 1.8 to 183 μg/g DW, respectively.

Very few studies reported microalgal vitamin B_5_ concentration, with a maximum of 190 μg/g DW measured in *Chlorella* (Table 4). In the same species, high vitamin B_9_ concentration was also revealed (from 3.1 to 34 μg/g DW). High vitamin B_9_ content was described in *Picochlorum sp.* (64.7 μg/g DW) and *Michrochloropsis* (43.6 μg/g DW). The range of variability of vitamin B_9_ content was greater in green algae (from 0.4 to 64.7 μg/g DW) than in cyanobacteria (0.27 to 15 μg/g DW). Vitamin B_7_ content ranged between 0.18 and 1.9 ng/g DW (Table 4) with the highest values reported in *Stichococcus* and *Tetraselmis* (1.3 ng/g DW), *Nannochloropsis* (1.1 ng/g DW) and in the haptophycean *Pavlova* (1.9 ng/g DW).

### Vitamins D and K content

Microalgae can contain a high concentration of the two forms of vitamin D (D_2_ and D_3_, [97], Table 5) and represent the main source of these vitamins for fish, which is one of the major providers of vitamin D for humans [10].

**Table 5:** Vitamin D content in microalgae

Phylum/class	Species	Vitamin D	References
Cyanobacteria	*Arthrospira maxima*	0.004	[22]
Chlorophyta	*Chlorella minutissima*	0.004	[22]
	*Tetraselmis sp. CS-362*	0.35	[77]
	*Tetraselmis suecica*	14	[78]
	*Stichococcus sp. CS-92*	0.35	[77]
Rhodophyta	*Rhodomonas salina*	0.004	[22]
Bacillariophyceae	*Skeletonema costatum*	11	[78]
Haptophyta	*Isochrysis galbana*	5	[78]
	*Pavlova lutheri*	39	[78]
	*Pavlova pinguis*	0.35	[77]
Eustigmatophyceae	*Nannochloropsis sp. CS-246*	0.35	[77]
	*Nannochloropsis oceanica*	0.48	[22]

Values are expressed as µg/g DW

Very high concentration of vitamin D was reported in *Pavlov*a *lutheri* (39 µg/g DW), *Tetraselmis suecica* (14 µg/g DW) and *Skeletonema costatum* (11 µg/g DW).

Conversely, vitamin D concentration was very low (0.004 µg/g DW) in other species, such as *Rhodomonas salina*, *Arthrospira maxima* or *Chlorella minutissima* (Table 5). Also, ergosterol, precursor of vitamin D_2_, was found in various species of microalgae, e.g., *Dunaliella tertiolecta* [98], *Chlamydomonas reinhardtii* [35, 99], *Chlorella vulgaris* [37], *Cyanidium caldarium* [38] and account up to 0.1% of the dry weight in the coccolitophore *Emiliania huxleyi* [21].

Vitamin K was also higher in marine photosynthetic organisms than in terrestrial plants [7]. Vitamin K_1_ and vitamin K_2_ are unevenly distributed among algal divisions. Vitamin K_1_ concentration in microalgae ranged from 0.1 µg/g DW in the green microalga *Dunaliella salina* to 200.25 µg/g DW in the cyanobacterium *Anabaena cylindrica* (Table 6). High value (28 µg/g DW) was also found in the green microalga *Tetraselmis suecica*. Conversely, low values were reported in the haptophyceans *Pavlova lutheri*, *Isochrysis galbana* and in the cyanobacterium *Arthrospira* (6.5, 8 and 12.7 µg/g DW, respectively; Table 6). Although vitamin K_1_ was reported in *Skeletonema costatum* (5.5 µg/g DW), its presence was not revealed in other diatoms such as *Phaeodactylum* and *Chaetoceros* [78, 94]. To date, vitamin K_2_ has been reported in the red microalgae *Porphyridium purpureum* and *Cyanidium caldarium* [100], in the diatom *Chaetoceros gracilis* [101], as well as in the cyanobacteria *Gloeobacter violaceus* [102] and *Synechococcus sp*. [103]. Vitamin K_2_ content was generally reported as unit per photosystem; in the red microalga *Cyanidium* and in the cyanobacterium *Gloeobacter* two molecules of menaquinone per one molecule of chlorophyll have been found [100].

**Table 6:** Vitamin K_1_ content in microalgae

Phylum/class	Species	Vitamin K_1_	References
Cyanobacteria	*Anabaena cylindrica*	200.25	[7]
	*Arthrospira*	12.7	[7]
Chlorophyta	*Chlorella vulgaris*	0.73	[7]
	*Desmodesmus asymmetricus*	0.46	[7]
	*Dunaliella salina*	0.1	[7]
	*Tetraselmis suecica*	28	[78]
Bacillariophyceae	*Skeletonema costatum*	5.5	[78]
Haptophyta	*Isochrysis galbana*	8	[78]
	*Pavlova lutheri*	6.5	[78]
Eustigmatophyceae	*Nannochloropsis oculata*	0.17	[78]

Values are expressed as µg/g DW

### Vitamins’ content modulation in microalgae

**Fig. 2:**
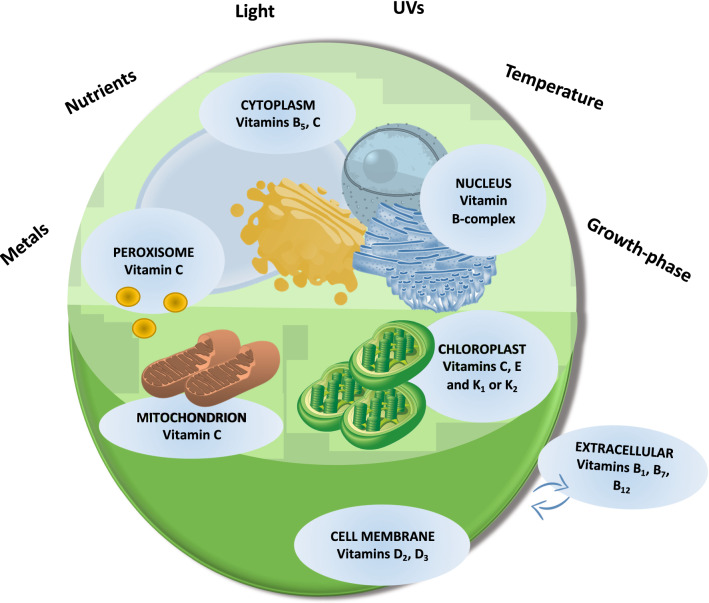
Intracellular location of vitamins in microalgae, and the environmental factors mainly modulating their content

Since vitamins are often used by photosynthetic organisms to regulate vital functions, their modulation in response to environmental changes is noteworthy; and this knowledge might be an important key for increasing vitamins production in microalgae. Many external parameters can affect vitamin synthesis and/or use in microalgae, namely light, temperature, salinity, nutrient or metal concentrations (Fig. 2), as well as cell density and growth stage. However, some vitamins are little investigated compared to others, e.g., vitamins C, A (pro-vitamin A = β-carotene) and E (α-tocopherol). Yet, less information is available on the vitamins’ content modulation in microalgae, compared to macroalgae. For instance, the brown macroalga *Eisenia arborea* modulates its vitamin pool content along with the seasonality, with the highest amount of vitamins A, B_1_, B_2_ and C revealed in spring in parallel with the lowest content of vitamin E [104]. Also, in the red macroalga *Palmaria palmata*, the provitamin A (β-carotene) increased during summer and lowered during winter [105]. Although seasonal variability of vitamins’ content in microalgae was not reported, their modulation by environmental changes were investigated in different studies. Light is known as strongly triggering bioactive compounds variations in microalgae [106]. For instance, vitamin E enhanced with increasing light intensity [79, 87, 107]. Similarly, in the cyanobacterium *Synechocystis* sp.PCC 6803, high light intensity increased the concentration of α-tocopherol [108]. Light intensity and spectral properties have been shown to significantly modulate ascorbic acid production and/or use in the coastal diatom *Skeletonema marinoi* [12, 13]. Vitamins content in microalgae is also affected by UV radiations. For instance, in the green alga *Chlorella vulgaris* vitamin E increased in presence of UV-B [109] while ascorbic acid did not [110]. Also, *Nannochloropsis oceanica* enhanced vitamin D_3_ in presence of UV-B, in a dose–response dependent manner, whereas no UV-B modulation of D_3_ concentration was recorded in other microalgae such as *Rhodomonas salina*, *Chlorella minutissima* or *Arthrospira maxima* [22].

The effect of temperature on vitamins production was poorly investigated. A seven-fold increase of α-tocopherol production was reported in *Euglena gracilis* under low temperature acclimation and oxygen stress [111].

Salinity variations induced vitamin B_1_ accumulation in microalgae such as *Nodularia spumigena* (cyanobacteria), *Phaeodactylum tricornutum, Skeletonema costatum* (diatoms), *Dunaliella tertiolecta* (chlorophyta), *Prorocentrum minimum* (dinoflagellate) and *Rhodomonas salina* (cryptophyte) [47].

Chemical variations of the cultivation environment also do affect vitamins production. Nutrient depletion enhanced the production of vitamins C and E in *Chlorella vulgaris, Tetraselmis suecica* and in the diatom *Phaeodactylum tricornutum* [26]. Similarly, α-tocopherol accumulated along with nitrogen concentration decrease in *Nannochloropsis oculata* [90], or with the addition of nitrate and phosphate in *Tetraselmis suecica* [87].

Pollutants such as heavy metals might also have effects on vitamins production or utilization in microalgae. For instance, the green alga *Scenedesmus quadricauda* lowered ascorbic acid to increasing heavy metals concentration [28]. Conversely, increased availability of cobalt chloride increased vitamin B_12_ concentration in *Chlorella vulgaris* [112]. Biological modulation of vitamins content in microalgae was also related to the growth phase (actively growing *vs.* stationary phase). The content of vitamin B_2_ increased by 2- to 3-folds in the stationary phase compared to the exponential phase in many microalgae (e.g., *Chaetoceros gracilis, Thalassiosira pseudonana, Isochrysis* sp., *Pavlova lutheri, Nannochloris atomus* or *Nannochloropsis oculate* [83]. Also, vitamin B_1_ enhanced during stationary phase in *Nannochloris atomus*, *Nannochloropsis oculata*, *Isochrysis* sp. and *Pavlova lutheri* [77], as well as in the diatoms *Chaetoceros muelleri*, *Thalassiosira pseudonana* [77]. and *Nitzschia microcephala* [113]. In *Chlorella ellipsoidea*, the vitamins B_1_, B_2_, B_6_ and B_9_ were more produced during the stationary phase of growth, while vitamins C, B_3_ and B_7_ were mainly synthetized during the active growth phase [114]. However, vitamin C modulation by growth phase is highly variable within microalgae [13, 77], and probably related to the biochemical function of ascorbic acid in cells [13].

### ***Vitamins B***_***12***_***, B***_***7***_*** and B***_***1***_*** auxotrophy in microalgae***

As pointed out recently [115], microalgae can be auxotrophs for the vitamins B_12_, B_7_ and B_1_. Among 306 microalgal species surveyed [116], more than half required vitamin B_12_ (cobalamin), while 22% required B_1_ (thiamine) and 5% required B_7_ (biotin), revealing that auxotrophy is shared by many species from unrelated classes (e.g., dinophyceae, raphidophyceae, bacillariophyceae, cryptophyceae and prymnesiophyceae). For instance, *Gymnodinium brevis* requires all three vitamins whereas *Gymnodinium spendens* requires only vitamin B_12_ [1]. Auxotrophy for B_12_ is ubiquitous in the haptophyte lineage [117], as in the coccolithophore *Emiliania huxleyi* [118], while a high variability is noteworthy in other classes. Some species can overcome B_12_ limitation in the environment thanks to a B_12_-independent methionine synthesis enzyme (e.g., *Chlorella sp.* NC64A, *Phaeodactylum tricornutum* CCAP1055/1, *Ectocarpus siliculosus* Ec32; (Katherine E [119]). Some microalgae (e.g., cyanobacteria, (Katherine Emma [120, 121]) are able to synthetize pseudocobalamin, which can be transformed into vitamin B_12_, the latter being more bioavailable (Katherine Emma [120, 121].

Vitamin B_1_ auxotrophy is diffused in marine microalgae [122], e.g. 80% of prymnesiophytes [1, 123] although with a lower percentage in diatoms [123]. Interestingly, thiamine biosynthesis in some microalgae (e.g., *Chlamydomonas reinhardtii*) can be induced and even regulated thanks to a riboswitch process regarding the gene encoding for the enzymes involved in thiamine biosynthesis [124] activated by the presence of thiamine in the environment. Microalgae can therefore become performant producers of thiamine [47].

Although some algae are auxotrophs for biotin, the ability to produce this molecule is transversally present in diverse microalgal classes, as shown by a genome-wide analysis performed on 14 photosynthetic microalgae (10 Chlorophyta, 1 Rhodophyta; 1 Haptophyta and 2 Heterokontophyta) that revealed the presence of a bifunctional enzyme involved in vitamin B_7_ (biotin) production [125].

## Vitamins and human health

Although vitamins are not structural components, and required by cells in low amount, they are essential for life, growth and development. Vitamins participate to cell homeostasis and to anabolic pathways as enzymatic cofactors. Humans are not able to endogenously synthesize adequate concentrations of vitamins for the normal physiological functions requiring their exogenous intake through foods and dietary supplements. Indexes such as Adequate Intakes (AI) and Recommended Dietary Allowances (RDA) were provided (Table 7; modified from [126]).

**Table 7:** Recommended Dietary Allowances (RDA) and Adequate Intakes (AI, values with *)

	Vit. A (μg/day)	Vit.C (mg/day)	Vit.D (μg/day)	Vit.E (mg/day)	Vit.K (μg/day)	Vit.B_1_ (mg/day)	Vit.B_2_ (mg/day)	Vit.B_3_ (mg/day)	Vit.B_5_ (mg/day)	Vit.B_6_ (mg/day)	Vit.B_7_ (μg/day)	Vit.B_9_ (μg/day)	Vit.B_12_ (μg/day)
Infants (months)													
0–6	400*	40*	10*	4*	2.0*	0.2*	0.3*	2*	1.7*	0.1*	5*	65*	0.4*
6–12 m	500*	50*	10*	5*	2.5*	0.3*	0.4*	4*	1.8*	0.3*	6*	80*	0.5*
Children (years)													
1–3	300	15	15	6	30*	0.5	0.5	6	2*	0.5	8*	150	0.9
4–8	400	25	15	7	55*	0.6	0.6	8	3*	0.6	12*	200	1.2
Males (years)													
9–13	600	45	15	11	60*	0.9	0.9	12	4*	1.0	20*	300	1.8
14–18	900	75	15	15	75*	1.2	1.3	16	5*	1.3	25*	400	2.4
19–30	900	90	15	15	120*	1.2	1.3	16	5*	1.3	30*	400	2.4
31–50	900	90	15	15	120*	1.2	1.3	16	5*	1.3	30*	400	2.4
51–70y	900	90	15	15	120*	1.2	1.3	16	5*	1.7	30*	400	2.4
> 70	900	90	20	15	120*	1.2	1.3	16	5*	1.7	30*	400	2.4
Females													
9–13	600	45	15	11	60*	0.9	0.9	12	4*	1.0	20*	300	1.8
14–18	700	65	15	15	75*	1.0	1.0	14	5*	1.2	25*	400	2.4
19–30	700	75	15	15	90*	1.1	1.1	14	5*	1.3	30*	400	2.4
31–50	700	75	15	15	90*	1.1	1.1	14	5*	1.3	30*	400	2.4
51–70	700	75	15	15	90*	1.1	1.1	14	5*	1.5	30*	400	2.4
> 70	700	75	20	15	90*	1.1	1.1	14	5*	1.5	30*	400	2.4

RDA represents the average daily dietary intake level sufficient to meet the nutrient requirements of nearly all healthy individuals. It is calculated from an Estimated Average Requirement (EAR). If sufficient scientific evidence is not available to establish an EAR, an AI is usually developed.

Fat-soluble vitamins are absorbed through the intestinal tract with the help of lipids (fats), and can be retained for long periods of time in the body while if consumed in excess can pose a greater risk for toxicity than water-soluble vitamins.

Water-soluble vitamins dissolve easily in water, so consistent daily intake is often required, being easily excreted and not stored in the body. In addition, water soluble vitamins are difficult to preserve during food storage and preparation because readily destroyed or washed out.

**Vitamin A** is essential for embryonic development, tissues differentiation, growth, epithelial integrity, red blood cell production, reproduction, immune function, and the visual system [127]. Retinol functions as an electron carrier in mitochondria [128] and is the precursor of bioactive retinaldehyde and retinoic acid. Vitamin A derivatives have dual functions in physiology: 11-cis-Retinal serves as the universal chromophore of the visual pigments in the eye, whereas retinoic acid regulates the expression of target genes via activation of two classes of nuclear receptors, the retinoic acid receptors and the retinoid X receptors [129]. Deficiency in vitamin A is one of the major factors implicated in the pathogenesis of anaemia. During pregnancy, an additional intake of vitamin A is recommended by the World Health Organization (WHO) in developing countries, for the prevention of night blindness, without exceeding in consumption for its teratogenic side effect and for the increased risk of vomiting and fontanel bulging observed in trials testing therapeutic doses among infants. Many studies conducted among populations deficient in vitamin A, revealed that vitamin A reduces diarrhoea-related mortality (28%) and new episodes of diarrhoea (15%). Concerning chemoprevention strategy for cardiovascular and cancer diseases, some epidemiological and clinical trial studies [130] revealed an increase in lung cancer incidence for patients (mainly smokers) that have supplemented their diet with vitamin A in combination with β-carotene in last five years before diagnosis.

**Vitamin D** regulates calcium and phosphate metabolism, so it is responsible for the formation and maintenance of bones. It is related to the postmenopausal women health, with particular attention to the fracture prevention in the case of osteoporosis disease [131]. Another important role of vitamin D in good health status maintenance regards the correct intake of it during pregnancy for the prevention of low birth-weight and preterm delivery [132]. Vitamin D and its analogues may be effective in preventing many types of human cancer diseases including breast cancer, prostate cancer, colorectal cancer, and some hematological malignances [133]. Most recent finding about vitamin D bioactivity regards its role in the prevention of COVID19 infection and mortality [134]. A relationship between vitamin D presence and the reduction of complications in COVID19 patients attributed to downregulated inflammation and cytokine production has been highlighted [135].

**Vitamin E** role is mainly based on its antioxidant properties, especially in prevention [136]. of lipid peroxidation and oxidative stress related diseases especially in epithelial tissues [137]. These pathological conditions include cardiovascular diseases, cancers, cataracts, macular degeneration, and neurodegenerative diseases such as Alzheimer disease [138].

**Vitamin K** is a key regulator for the synthesis of blood clotting factors in the liver: It is associated with disorders mainly related to coagulation. In particular, vitamin K deficiency is also linked to other pathological conditions, such as malabsorption disorders, antibiotics and drug interactions, especially with coumarin-based anticoagulants [139].

**Vitamin C** is an essential dietary component for human nutrition, being a strong antioxidant and exerting an immunostimulant and chemopreventive function. Deficiency of vitamin C causes “scurvy” with severe symptoms such as impaired wound healing, hemorrhage and edema, commonly manifest as swollen bleeding gums [140]. Unfortunately, vitamin C is one of the most unstable nutrients in presence of oxygen, metal ions, increased pH, heat or light [141]. In fact, cooking processes and long-term storage determine a significant loss of vitamin C [142]. Another important bioactivity of vitamin C concerns its role against chronic and acute diseases mainly related to oxidative stress such as cancer, cardiovascular disease [143], hypertension, stroke [143], and neurodegenerative disorder [144, 145].

**Vitamin B**_**1**_—thiamine pyrophosphate is the metabolically functional form—is a nitrogen containing catalyst which plays a major role in glycolysis [115]. Vitamin B_1_ has a key role in the synthesis of neurotransmitters and in the correct function of the neural system [146]. Deficiency in vitamin B_1_ causes syndromes such as beriberi, polyneuritis, and Wernicke-Korsakoff. The primary symptoms of this vitamin lack include severe decreases in appetite, in growth, bradycardia, and muscular weakness.

**Vitamin B**_**2**_ (riboflavin) functions as a catalyst for redox reactions in numerous metabolic pathways and in energy production [140]. The active forms of vitamin B_2_ are cofactors for enzymatic reactions in the TCA cycle and in fatty acid oxidization [147]. Vitamin B_2_ has also role in chemoprevention of cancer and infective diseases due to its involvement in redox and photoreactions with nucleic acids for the inactivation and destruction of host cells [148, 149]. Another crucial role of vitamin B_2_ is the involvement in the metabolism of vitamins B_6_, B_9_ and B_12_ and its deficiency determines an insufficient recruitment of these other vitamins [150, 151]. Also, deficiency states in vitamin B_2_ generate various symptoms such as loss of appetite and depressed growth, cheilosis, angular stomatitis, and dermatitis, at neural level ataxia and paralysis, and vascular disorders.

**Vitamin B**_**3**_ (niacin) can be synthesized by mammals via an endogenous enzymatic pathway from tryptophan and is stored in the liver [152]. Vitamin B_3_ is also synthesized from tryptophan by intestinal bacteria [153, 154]. In the form of the coenzymes NAD and NADP, niacin functions in many biological redox reactions. Niacin deficiency affects many organs, such as skin inflammation with exposure to sunlight becoming pathology well known as pellagra. Pellagra includes other symptoms such as diarrhea, depression or dementia [155]. In some cases, it was observed that niacin deficiency is also associated with schizophrenia [156]. Niacin is metabolically synthetized from the amino acid tryptophan with a ratio of 1 mg of dietary niacin for 60 mg of tryptophan [157].

**Vitamin B**_**5**_ (pantothenic acid) has a potential cardioprotective role exerting anti-inflammatory effects through antioxidant properties [5]. Pantothenic acid deficiency although rare, causes dangerous effects on the liver (e.g., steatosis) and the nervous system (e.g., paralysis), together with a-specific symptoms such as decreased appetite and fatigue [140]. Pantethine, a disulphide form of panthothenic acid, is synthesized in the body and considered as the most active form of vitamin B_5_ due to its sulfhydryl-group [158].

**Vitamin B**_**6**_ is widely distributed in dietary sources and in addition synthetized by gut microflora [159]. Clinical deficiency of vitamin B_6_ generally occurs together with all vitamin B complex [160]. In particular there are cases of vitamin B_6_ deficiency, such as anemia post pancreaticoduodenectomy [161]. Vitamin B_6_ contributes to fatty acid biosynthesis, breakdown of certain storage compounds as well as in the biosynthesis of neurotransmitters [20, 162–167].

**Vitamin B**_**7**_ (biotin) is widely distributed in food items and synthesized in meaningful amounts by gut microflora in humans. Recently it was showed the role of biotin in immune-mediated intestinal inflammation [168].

**Vitamin B**_**9**_ is converted by intestinal bacteria into its active form tetrahydrofolate [169, 170] starting from folates, which are widely available in dietary sources of plant and animal origins [140]. Folates have important roles in various catabolic and biosynthetic routes through numerous reactions that involve, among the others DNA and purine synthesis [171]. Folates are also involved in amino acid and nucleotide metabolism and methylation reactions, thus having a fundamental role in normal embryogenesis by supporting cell division. For this reason, it is recommended to assume a correct dietary intake of folate during early pregnancy, in order to significantly reduce the risk of neural tube defects at birth [172]. Folate deficiency may cause impaired biosynthesis of DNA together with clinical symptoms of megaloblastic anemia, alopecia, achromotrichia, and neuropathy [173]. It is noteworthy that the bioavailability of naturally occurring folates is low if compared to synthetic folic acid, normally used in food fortification and supplements [174].

**Vitamin B**_**12**_ (cyanocobalamin) can be converted to either of the two important active forms: methylcobalamin and 5-deoxyadenosylcobalamin [175]. In humans, where it is required in trace amounts, B_12_ is a cofactor for two enzymes: methionine synthase and L-methylmalonyl-CoA mutase [71, 176]. These enzymes have crucial roles in amino acid and fatty acid metabolism, and DNA synthesis. Methionine synthase also requires folate for its action. Vitamin B_12_ is widely distributed in human food of animal or vegetable origin, such as edible algae and fermented soybean-based foods [177]. Deficiency in vitamin B_12_ might induce peripheral neuropathy and neurological dysfunction (e.g., cognition) [140] and, when associated with folate depletion, it becomes one of the main causes of megaloblastic anemia [178].

## Microalgal vitamins and human health

Algal foods offer one of the few vegetarian alternatives for cobalamin in the diet. While some studies hypothesized that algal-derived vitamin B_12_ was not bioavailable to humans [179], other authors showed that increased consumption of *Chlorella* or nori by vegan people prevented B_12_ deficiency [177]. Also, feeding nori to vitamin B_12_-deficient rats yielded a 1.9-fold increase in hepatic levels of total B_12_ compared to those without nori supplementation [180]. Therefore, algal foods offer one of the few vegetarian alternatives for cobalamin in the diet [181].

Among microalgae, *Spirulina* is called “superfood” [182] thanks to its richness in vitamins (A, E, K, B_1_, B_2_, B_3_, B_6_ and B_12_) together with its macromolecular composition, in term of proteins and other bioactive compounds [5, 182]. One g of commercial *Spirulina* powder supplies up to half of the RDA for β-carotene and vitamin B_12_ [183], with a recommended consumption of less than 4 g per day for an average healthy adult to avoid any toxic effect [184]. Yet, *Chlorella pyrenoidosa* powder reduced the risk of anemia, proteinuria and edema in pregnant women [185] thanks to its high content in thiamine, riboflavin, folic acid, and biotin [186].

One of the main sources of vitamin D is represented indirectly by (micro)algae, that which ingested by seafood, allow them to provide vitamin D to humans. The direct use of micro(algae) in this context would increase the efficiency and meet with the vegetarian or vegan requirements.

The microalgal production related industry is currently increasing as the global nutraceutical market size is projected to reach USD 722.49 billion by 2027 [187]. Vitamins and minerals together accounted for over 40.71% share in 2019 while functional food accounted for the largest share in 2019 and generated revenue of USD 187.51 billion [187].

Algal species of *Nannochloropsis* and *Chlorella vulgaris* are primary ingredients used in the sport nutrition industry and are priced at about USD 18,000–36,000 t^−1^ [187]. For instance, *Chlorella* is one of the top-selling food supplements in Japan and it is produced by > 70 companies worldwide [188, 189]. Also, β-carotene from *Dunaliella* currently values USD 1500 per kilogram, and its use as a nontoxic vitamin A precursor has made it a mainstay in multivitamin and specialty formulations [190].

**Fig. 3:**
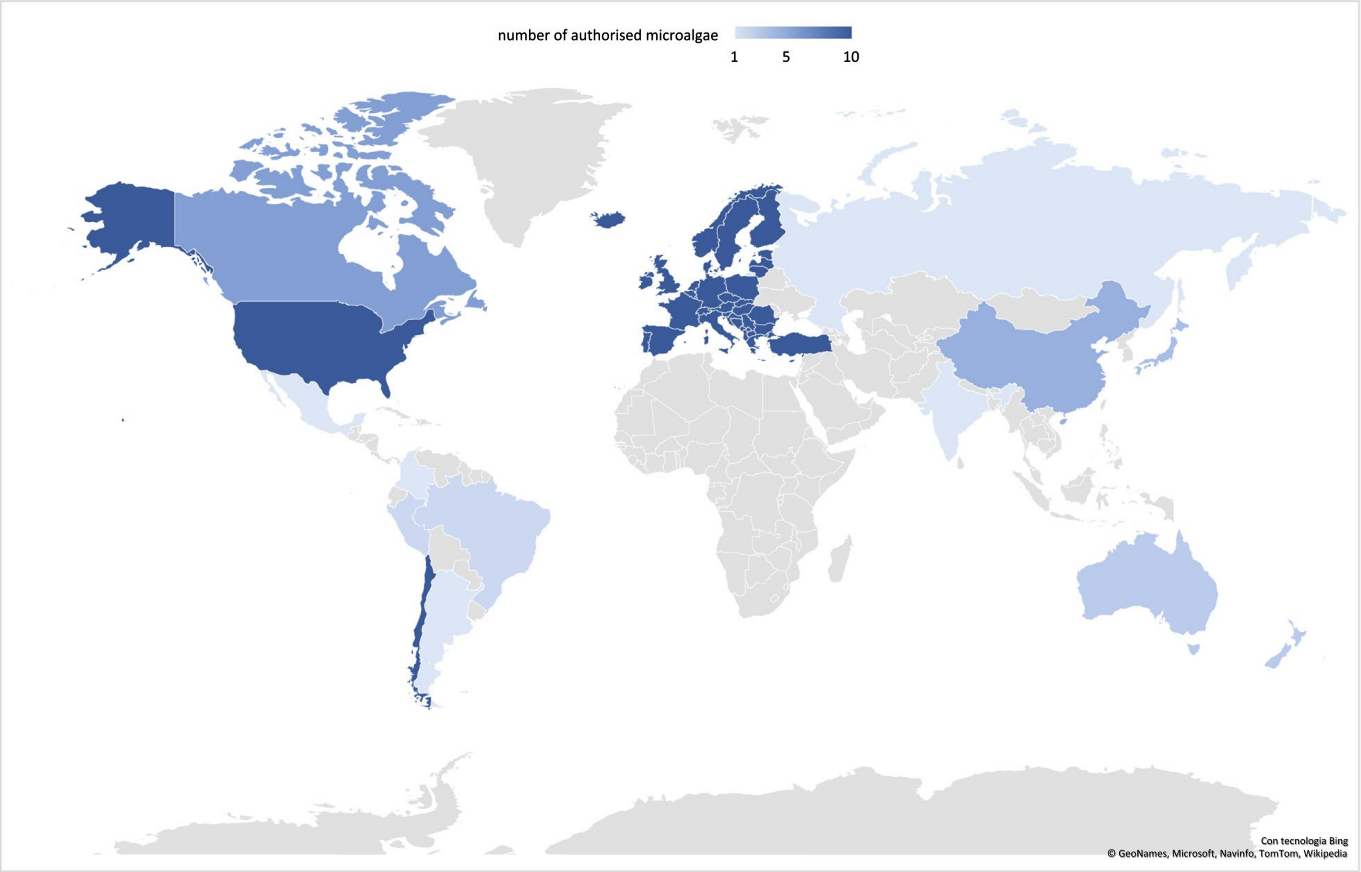
Map of the number of authorised microalgae species worldwide for direct human consumption. In grey, absence of data. (Data sources: https://www.argentina.gob.ar/anmat/codigoalimentario (Argentina); https://www.foodstandards.gov.au/Pages/default.aspx (Australia and New Zealand); https://portal.anvisa.gov.br (Brasil); https://health-products.canada.ca/lnhpd-bdpsnh/index-eng.jsp (Canada); https://www.fia.cl/wp-content/uploads/2018/03/N-3-Revista-Mayo-2016.pdf (Chile); https://en.nhc.gov.cn/2018-10/22/c_74485.htm (China); https://www.invima.gov.co (Colombia); https://old.fssai.gov.in/GazettedNotifications.aspx (India); https://www.jetro.go.jp/ext_images/en/reports/regulations/pdf/foodext2010e.pdf (Japan); https://www.gob.mx/cofepris (Mexico); https://www.ins.gob.pe/insvirtual/images/otrpubs/pdf/Tabla%20de%20Alimentos.pdf (Peru); https://patents.google.com/patent/RU2137402C1/en (Russia); https://ec.europa.eu/food/safety/novel_food/catalogue/search/public/index.cfm (UE and observers); https://www.fda.gov/food/generally-recognized-safe-gras/gras-notice-inventory (USA))

In EU, under the European Food Safety Authority (EFSA) (Regulation ECNo 2015/2283 [191] several microalgae are authorized as food products (Fig. 3), including *Anabaena flos-aquae, Arthrospira platensis, Chlorella luteoviridis, Chlorella pyrenoidosa, Chlorella vulgaris, Odontella aurita, Tetraselmis chui* and astaxanthin from *Haematococcus pluvialis*. In USA, the Food and Drug Administration [191, 192] currently recognizes few microalgae as safe for human consumption (Fig. 3), namely *Arthrospira platensis, Chlamydomonas reinhardtii, Auxenochlorella protothecoides, Chlorella vulgaris, Dunaliella bardawil* and *Euglena gracilis* [189]. While *Arthrospira platensis* is currently used as food worldwide (Canada, China, EU, India, and Japan), the other species vary with the geographical areas (*Chlorella protothecoides* in the U.S. and Japan, *C. pyrenoide*sa in EU and China, *C. vulgaris* in Canada, EU and Japan, etc. [189]). Also, it has to be noted that all these microalgae belong to cyanobacteria or green algae groups, except *O. aurita* which is the unique diatom in this regulated panorama.

Studies combining the analysis of vitamin concentrations together with testing algal product as food complements or functional food are needed to enhance the role of microalgae as food complements [180]. Also, the evaluation of the digestibility of microalgal biomass is required. In vitro models simulating human digestion are used to assess structural changes, digestibility and release of food components [193, 194], e.g. evaluating several seaweeds and microalgae food products, which highlighted class-related differences [192, 195–197]. Eukaryotic microalgae can present a robust multi-layered cell wall in which cellulose, hemicellulose, pectin compounds, glycoproteins and algaenan can limit the access of the digestive enzymes to the cell components. Conversely, cyanobacteria appear to be more easily digestible due to their peptidoglycan layer and the proteic and lipopolysaccharidic outer membrane [192, 198].

The relationship between vitamins content and human health or wellness is not direct. The bioaccessibility and bioavailability of vitamins are different amongst vitamins and foods. Also, they are not all absorbed/retained in the same way. Furthermore, synergy between different bioactive compounds might enhance their beneficial effects [199]. For instance, the effectiveness of carotenoids as antioxidants is dependent upon their interaction with other co-antioxidants, especially vitamins E and C [200, 201]. Vitamin C acts as a potent synergist in the presence of α-tocopherol enhancing its antioxidant activity [201, 202]. This effect can be further enhanced by phenolic compounds such as quercetin, forming a non-covalent association at the cytosol-membrane interface within the lipid bilayer in membranes, originating a complex in which antioxidant regeneration is significantly enhanced [203].

## Microalgal challenges for vitamin production

**Fig. 4:**
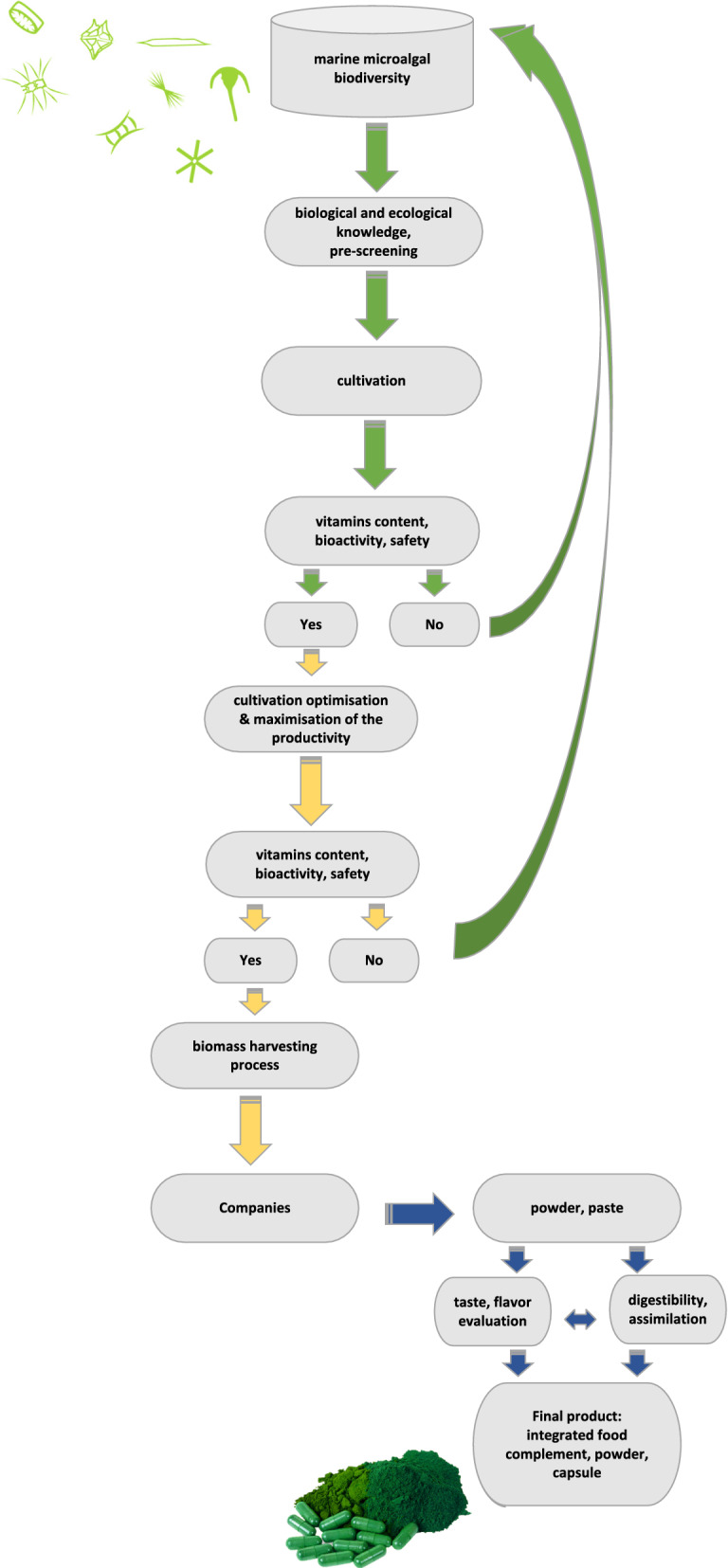
Pipeline design: Research and development strategies for improving microalgal vitamins uses for human food

Environmental manipulations can be a low-cost and effective way to modulate biosynthetic pathways and the natural production of vitamins enriched microalgal biomass, starting from the optimization of the resonance between growth, ecophysiological requirements and the environmental/cultivation climate (Fig. 4). Attempts regarding enhancing microalgal vitamins production were already carried out. Optimization of α-tocopherol production has been done with *Euglena gracilis* Z, also maximizing β-carotene yield with mixotrophic cultivation [204]. UV-B light administration (until 4.4 kJ m^−2^) improved the production of α-tocopherol and β-carotene in *Chlorella vulgaris* [109]. Also, high α-tocopherol productivity was achieved in *Euglena* by modifying culture conditions [111] or through a two-step cultivation strategy [79, 107]. Two-step cultivation strategy was also carried out for enhancing β-carotene production in *Dunaliella* [98, 205]. They reported increased β-carotene productivity to 450 mg m^−2^ day^−1^ in stage one and to 300 mg m^−2^ day^−1^ in stage two, instead of the 200 mg β-carotene m^−2^ day^−1^ yield obtained via the conventional cultivation.

A significant increase of ascorbic acid production (0.79 mg ascorbic acid g^−1^) has also been obtained in *Tetraselmis* sp. cultivated in 100 m^3^ photobioreactors [181]. Light climate—spectrum and intensity—variations tuned up the production of ascorbic acid in the diatom *Skeletonema marinoi* [12, 13]. Heterotrophy is also a way for increasing productive the yield of ascorbic acid, as shown in the red microalga *Galdieria partita* [206]. Heterotrophic synthesis of L-ascorbic acid has been also performed in the green microalga *Chlorella pyrenoidosa* [207]. Production of vitamin K_1_ of 40 μg L^−1^ day^−1^ was achieved by cultivating the cyanobacteria *Anabaena cylindrical* varying the medium composition and day length [94]. Vitamin D_3_ accumulation might be obtained thanks to interactions between microalga and UV-B light as revealed in the eustigmatophycean *Nannochloropsis* sp. [22]. The addition of cobalt chloride salt in Bold’s Basal Medium maximized vitamin B_12_ content in *Chlorella vulgaris* with a 7–12% higher content than control condition [112]. Also, for vitamins B_1_ and B_2_, the tuning of light could increase the production of vitamins B_1_ and B_2_ [77, 83].

Although physical or chemical manipulation of cultivation techniques is one way to improve the yield of vitamin production per microalgal biomass unit, biological manipulation might be undertaken. Some attempts of genetic manipulations of microalgae for enhancing bioactive compounds production (e.g., vitamins) are also on-going. However, this route does not ensure the maintenance of optimal growth of such organisms and poses the question of “genetically modified organisms” whose entrance into the food market could be extremely difficult. Nuclear transformants of the green model alga *Chlamydomonas reinhardtii* expressing protein intrinsic factors have been generated, suggesting that microalgae can represent a viable host for the production of a vegetarian protein intrinsic factor, source for B_12_ enrichment [208]. Also, the potential of riboswitches in microalgae [209] might be of interest for genetic manipulations aiming to enhanced thiamine production.

Another route of biological manipulation for vitamins’ productive yield increase is the co-cultivation between at least two different species (alga-bacteria or alga-alga; [15]). Bacteria-microalga co-cultivation might improve the yield of harvested microalgal biomass [101], and a way to protect microalgae against pathogens through the synthesis of antibiotics from bacteria as well as to enhance the synthesis of microalgal specific compounds. Vitamins can be a target for such strategy, especially concerning vitamins B supply since microalgae are mainly auxotrophs for some of them [1, 15, 115, 210]. Different studies showed the interests of the mutualistic relationships between microalgae and bacteria, the latter providing vitamin B_12_ [211]. Interests of co-cultivation for thiamine production in some microalgae, e.g. *Chlamydomonas reinhardtii*, are linked to the capacity of these microalgae to activate the biosynthetic pathway of vitamin B_1_ by sensing the presence of vitamin B_1_ from outside, e.g. produced by bacteria [212]. For biotin (vitamin B_7_), results on mutualistic relationships between algae and other organisms (bacteria or fungus) are few [115, 213].

Microalga-microalga co-cultivation might be a real alternative, aiming to improve the yield synergetic bioactive compounds production. This strategy requires knowledge on species/genera/classes of microalgae and the selection of species enhancing mutualism or commensalism, avoiding parasitism or competition for the same resources, e.g. light spectrum (e.g., blue: green ratio), nitrogen source (nitrates, ammonium, organic nitrogen sources) or silica. This route focusses on the final harvested microalgal product more than on the functional mutualistic relationships between microalgae. The complementarity of microalgae in terms of nutritional values paves the way to investigate their integration in a unique cultivation step. For instance, diatoms, rich in carotenoids, polyphenols, some vitamins (e.g., A and C) and lipids can be mixed with cyanophytes, rich in proteins, vitamins (e.g., B) and phycobiliproteins, to provide a “super synergetic microalgal product”. Yet, a co-cultivation of small and big species might be a choice, small species having a lower level of requirements from outside than bigger species. Also, the co-cultivation of vitamins B producer alga and a non-vitamin B producer (with greater ability to synthesize other bioactive compounds) is a way to finally produce and high bioactive quality biomass. Research activities in this sense are on-going and the results highly promising (Brunet et al., personal communication).

All the aforementioned strategies could increase the yield of both the biomass and the molecules of interest. Prior microalgal utilization as functional ingredients or nutraceuticals, further investigation must be undertaken. Certain types of manipulation could imbalance microalgal nutritional values or even compromise their safety. Therefore, downstream studies assessing the safety and quality of the final product are mandatory.

## Conclusions

For humans, microalgae can be a source of vitamins, together with other compounds, which increase the bioactive and nutraceutical value of microalgal biomass. The biotechnological interest of microalgae relies on their small size, high growth rate, reduced space needed for cultivation, and richness in bioactive compounds [214–216]. Microalgae have the potential to fill many of the global demand regarding different fields (e.g., nutraceuticals, energy, animal feed) being considered as valuable biofactories [217]. Increasing literature assessed that microalgae cover antiviral, antitumor, antioxidant, anti-inflammatory, antiallergenic, antidiabetic, and antibacterial properties [218–220]. So far, the limitations of developing industrial microalgal biotechnology are mainly represented by the high production costs [221, 222]. Lowering costs require an optimization of all the steps from the microalgal species selection to the cultivation and biomass harvesting until the extraction and fractionation of products. Multidisciplinary integration of tools (bioinformatics, system biology, molecular biology; [223]) as well as artificial intelligence [224] might provide a synergy for a systems-level understanding of microalgal production, improving the output of industrially valuable strains. Moreover biological, physiological and ecological data need to be integrated to better develop the biotechnological pipeline (Fig. 4) from species chemo- or bio-diversity to its industrial up-scaling [14]. Indeed, the great biodiversity enhances the microalgal potential for the biotechnological production of high valuable molecules, such as vitamins. Thanks to the richness and diversity of vitamins present in microalgae, they are potentially one of the main targets for developing microalgal biotechnology.

## Data Availability

Data sharing is not applicable to this article as no datasets were generated or analysed during the current study.
